# Variants and variability – a cohort of Omani children sheds more light on autosomal recessive polycystic kidney disease

**DOI:** 10.1007/s00467-026-07490-5

**Published:** 2026-07-24

**Authors:** Kathrin Burgmaier, Max C. Liebau

**Affiliations:** 1 Department of Pediatrics, University Hospital Cologne and Faculty of Medicine, University of Cologne, Cologne, Germany; 2 Faculty of Applied Healthcare Science, Deggendorf Institute of Technology, Deggendorf, Germany; 3 Department of Pediatrics, University Hospital RWTH Aachen, Aachen, Germany

Treating patients with autosomal recessive polycystic kidney disease (ARPKD) remains a major challenge for pediatric nephrologists. ARPKD is caused by biallelic disease-causing variants in various genes, most frequently *PKHD1* (*polycystic kidney and hepatic disease 1*) [1, 2]. This complex systemic disease typically presents very early in life. However, there is marked clinical variability of disease courses and the underlying mechanisms remain incompletely understood. There is a clear need for a better understanding of the disease.

A recent report by Al Riyami et al. [3] in *Pediatric Nephrology* provides insight into the clinical spectrum, genetic variants and outcomes of 114 Omani children with ARPKD. In their work, Al Riyami et al. [3] report genetic findings in 81 individuals with a limited *PKHD1* variant spectrum encompassing missense variants in c.107C > T, c.4870C > T and c.406A > G. The authors first describe the general clinical course of their cohort. They find high proportions with parental consanguinity (64%), abnormal antenatal scans (44%) and presentation before 6 months of life (59%). In the total cohort of 114 individuals with a median follow-up time of 62 months and the median age at last follow-up of 8.7 years the authors describe systemic hypertension in 55%, portal hypertension in 20%, varices in 14%, chronic kidney disease in 60% and kidney failure in 12% of patients.

The authors then proceed to a more detailed study of genetically characterized patients. While c.107C > T constitutes the most frequently detected variant in ARPKD in cohorts described in Europe and the USA [4–7], the variant c.406A > G has been detected in the Omani population [8, 9] and may be specific to the Omani population. There are no reports of identified truncating variants in the cohort presented by Al Riyami et al. [3]. Most interestingly, the work identifies two subgroups of patients, one group with homozygous variants in c.107C > T in 33 individuals from 23 families and one with homozygous variants in c.4870C > T in 24 individuals from 21 families. These two subgroups are unique as they allow a deeper understanding of the genotype–phenotype correlations in two large cohorts with homozygous variants and add important insight to previous findings. The authors are to be congratulated for studying this important aspect.

It seems relevant that major outcomes occur more frequently in individuals with homozygous variants in c.107C > T than in c.4870C > T [3]. Major outcomes, namely onset of kidney replacement therapy (KRT), portal hypertension, presence of varices, gastrointestinal (GI) bleeding and death, were more frequently reported in the subgroup with homozygous variants in c.107C > T than in the subgroup with homozygous variants in c.4870C > T. The authors visualize the events in individual patients in both subgroups – and the reader can easily realize that there were no age-documented events during the observation period in the subgroup with homozygous variants in c.4870C > T for the endpoints: onset of KRT, varices, GI bleeding and death. Substantially distinct follow-up durations (median 6.0 [Q1–Q3 1.5–12.7] (range 0.0–13.6) years; n = 15 vs. 2.7 [1.2–6.5] (0.0–11.8) years; n = 21; p = 0.123) and ages at last visit (median 8.1 [Q1–Q3 2.1–13.1] (range 0.1–15.1) years; n = 31 vs. 4.3 [1.9–7.8] (0.8–13.0) years; n = 23; p = 0.083) in both subgroups need to be kept in mind, as these may result in a bias. Additionally, not all patients in the respective groups could be included in the detailed analyses. But even with these caveats in mind, and even if the reader focusses on the first 4 to 5 years (where the median age at last visit in the “shorter” subgroup lies), one can adopt the concept of fewer events in the “milder” subgroup (homozygous variants in c.4870C > T, affecting amino acid 1624) compared to the “more severe” subgroup (homozygous variants in c.107C > T, affecting amino acid 36) both in kidney and liver associated endpoints. This is in line with previous analyses [5, 7]. Matching this, the native kidney estimated glomerular filtration rate at ages 1 to 5 years trended higher in the subgroup with homozygous variants in c.4870C > T.

A second very remarkable observation in this report is the high clinical variability in individuals with homozygous variants in c.107C > T. There is an ongoing discussion about the role of disease-modifying factors in patients with ARPKD. Studies have been hampered by the high percentage of patients with compound heterozygous variants in the very large *PKHD1* gene in ARPKD and a wide distribution of disease-causing variants along *PKHD1*. A relatively large number of patients with homozygous variants in the most frequently affected position c.107C > T with variable clinical courses as reported by Al Riyami et al. therefore seems of particular interest [3]. Al Riyami et al. report onset of KRT in 11 of 33 patients (33%) with homozygous variants in c.107C > T, portal hypertension in 42%, varices in 39%, GI bleeding in 6% and death in 18% of patients. The timepoints of documentations range between 3–16 years in case of KRT, 1–12 years in portal hypertension, 1–13 years in varices, 6–11 years in GI bleeding and 0–17 years in case of death. Thus, the report by Al Riyami et al. describes pronounced phenotypic variability within a unique subcohort with a well-defined genetic background (Omani population with homozygous variants in c.107C > T) and a high proportion of consanguinity. These observations suggest caution in approaches to anticipate clinical courses even in patients with ‘identical’ homozygous variants in the most frequently affected position, namely c.107C > T. Additional genetic or non-genetic factors beyond the variant in *PKHD1* seem to be relevant. An interesting additional future analysis could characterize intrafamilial variability within the defined subgroups.

Taken together, the report by Al Riyami et al. [3] sheds novel light into long-discussed questions in ARPKD. It also raises the questions, e.g. about effects of specific homozygous missense *PKHD1* variants and other effects, such as variants in modifier genes or environmental effects, leading to relevant clinical variability that also has been described previously [5, 10, 11]. In addition, it supports the concept that specific missense variants are associated with variable phenotypes. While disease-causing *PKHD1* variants are known to influence the phenotype and the prognosis, it is important to bear in mind that all subgroup analyses, including the one by Al Riyami et al., do not allow for prediction in an individual family or patient.

Future work will have to address potential other effects like modifier genes and environmental effects to even more improve our understanding of the observed clinical variability.
